# Crystal structure of a dinuclear Co^II^ complex with bridging fluoride ligands: di-μ-fluorido-bis­{tris­[(6-methyl­pyridin-2-yl)meth­yl]amine}­dicobalt(II) bis­(tetra­fluorido­borate)

**DOI:** 10.1107/S1600536814021631

**Published:** 2014-10-04

**Authors:** Masataka Inomata, Yusaku Suenaga

**Affiliations:** a Department of Science, Kinki University, 3-4-1 Kowakae, Higashi-Osaka 577-8502, Japan

**Keywords:** crystal structure, dinuclear cobalt complex, fluoride bridge, high-spin Co^II^ complex, tripodal ligand, C—H⋯F hydrogen bonds

## Abstract

Reaction of Co(BF_4_)_2_·6H_2_O with tris­[(6-methyl­pyridin-2-yl)meth­yl]amiine in methanol results in a fluoride abstraction from BF_4_^−^, yielding the unexpected title compound, [Co_2_F_2_(C_21_H_24_N_4_)_2_](BF_4_)_2_. The complex cation consists of two inversion-related [Co(C_21_H_24_N_4_)]^2+^ moieties bridged by a pair of fluoride ligands. The Co^II^ cation is six-coordinated in a distorted octa­hedral geometry and forms a +II high-spin state. In the crystal, the complex cation and the BF_4_^−^ anion are connected by C—H⋯F hydrogen bonds, forming a three-dimensional network. An intra­molecular C—H⋯F hydrogen bond is also observed.

## Related literature   

For related fluoride-bridging structures, see: Dugan *et al.* (2012 ▶); Ding *et al.* (2009 ▶). For related metal complexes with tripodal ligands, see: Massoud *et al.* (2008 ▶); Zhu *et al.* (2009 ▶); Beni *et al.* (2008 ▶). 
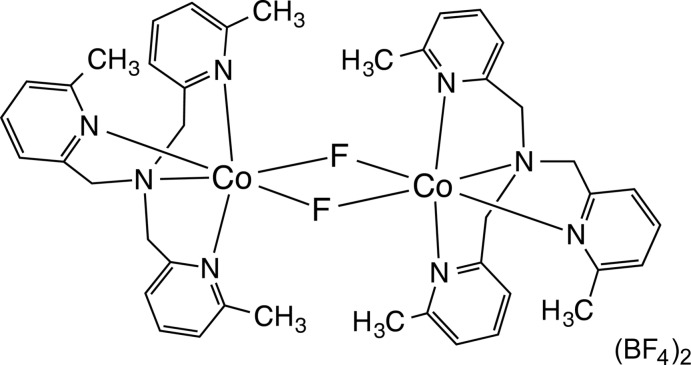


## Experimental   

### Crystal data  

[Co_2_F_2_(C_21_H_24_N_4_)_2_](BF_4_)_2_
*M* *_r_* = 994.36
Triclinic, 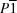

*a* = 8.7884 (17) Å
*b* = 11.334 (4) Å
*c* = 11.897 (2) Å
α = 64.91 (5)°
β = 82.04 (6)°
γ = 87.98 (7)°
*V* = 1062.5 (7) Å^3^
*Z* = 1
Mo *K*α radiation
μ = 0.87 mm^−1^
*T* = 120 K
0.20 × 0.20 × 0.10 mm

### Data collection  

Rigaku Mercury70 diffractometer
Absorption correction: multi-scan (*REQAB*; Rigaku, 1998 ▶) *T*_min_ = 0.787, *T*_max_ = 0.917
8221 measured reflections
4623 independent reflections
3660 reflections with *F* ^2^ > 2σ(*F* ^2^)
*R*_int_ = 0.033

### Refinement  

*R*[*F* ^2^ > 2σ(*F* ^2^)] = 0.053
*wR*(*F* ^2^) = 0.157
*S* = 1.01
4623 reflections
289 parameters
H-atom parameters constrained
Δρ_max_ = 0.98 e Å^−3^
Δρ_min_ = −0.62 e Å^−3^

### 

Data collection: *CrystalClear* (Rigaku, 2008 ▶); cell refinement: *CrystalClear*; data reduction: *CrystalClear*; program(s) used to solve structure: *SIR2011* (Burla *et al.*, 2012 ▶); program(s) used to refine structure: *SHELXL2013* (Sheldrick, 2008 ▶); molecular graphics: *CrystalStructure* (Rigaku, 2014 ▶); software used to prepare material for publication: *CrystalStructure*.

## Supplementary Material

Crystal structure: contains datablock(s) I. DOI: 10.1107/S1600536814021631/is5368sup1.cif

Structure factors: contains datablock(s) I. DOI: 10.1107/S1600536814021631/is5368Isup2.hkl

Supporting information file. DOI: 10.1107/S1600536814021631/is5368Isup3.doc

**Fig. 1.**
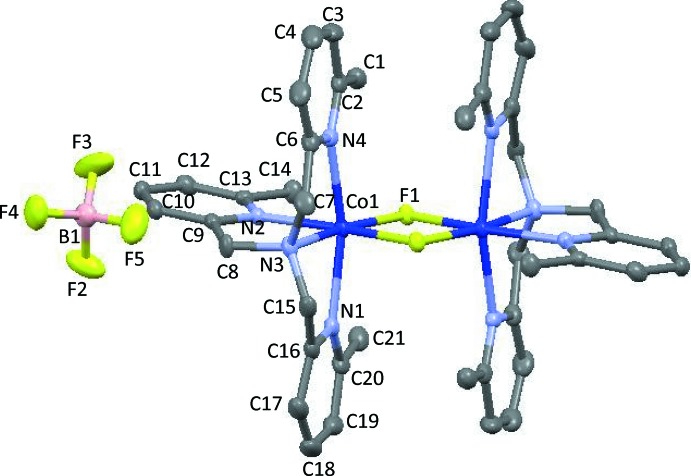
Perspective view of the complex showing 50% displacement ellipsoids. Hydrogen atoms are omitted for clarity.

. DOI: 10.1107/S1600536814021631/is5368fig1.tif
Perspective view of the complex showing 50% displacement ellipsoids. Hydrogen atoms are omitted for clarity.

CCDC reference: 1027138

Additional supporting information:  crystallographic information; 3D view; checkCIF report

## Figures and Tables

**Table 1:** Selected bond lengths ()

Co1F1	1.985(2)
Co1F1^i^	2.098(2)
Co1N1	2.249(3)
Co1N2	2.143(3)
Co1N3	2.124(3)
Co1N4	2.251(3)

Symmetry code: (i) 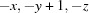.

**Table 2:** Hydrogen-bond geometry (, )

*D*H*A*	*D*H	H*A*	*D* *A*	*D*H*A*
C1H1*C*F1	0.98	2.37	3.298(5)	157
C3H3F3^ii^	0.95	2.43	3.296(5)	152
C5H5F5^iii^	0.95	2.46	3.231(6)	139
C10H10F2	0.95	2.52	3.372(5)	149
C10H10F3	0.95	2.53	3.294(5)	138
C12H12F2^iv^	0.95	2.51	3.327(5)	143
C14H14*A*F1	0.98	2.26	3.220(4)	167
C14H14*C*F2^v^	0.98	2.36	3.287(4)	157

Symmetry codes: (ii) 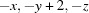; (iii) 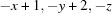; (iv) 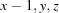; (v) 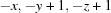.

## supplementary crystallographic information

### S1. Experimental

A solution of Co(BF_4_)_2_·6H_2_O (204 mg, 0.60 mmol) in dry methanol (20 ml) was added to a methanol solution (20 ml) of tris[(6-methylpyridin-2-yl)methyl]amiine (199 mg, 0.60 mmol). The resulting solution was stirred for 1 hr. Diethylether was added to the filtrate slowly to obtain the complex. This solution stand at ambient temperature and over the period of 7 days a purple microcrystals of [Co_2_(Me_3_tpa)_2_F_2_](BF_4_)_2_ separated from the solution in 18% (107 mg) yield. IR (KBr, cm^-1^); 3448 s, 1605 s, 1578*m*, 1451 s, 1352*m*, 1084 s, 789*m*, 522*m*, ESI-MS; m/*z*=907.26(M—BF_4_), Anal. Calc. for C_42_H_48_N_8_F_2_Co_2_B_2_F_8_: C 50.73, H 4.87, N 11.27%. Found: C 50.96, H 4.63, N 11.07%.

### S2. Refinement

H atoms were treated as riding, with C—H = 0.95 or 0.98 Å and with *U*_iso_(H) = 1.2*U*_eq_(C).

### Crystal data

**Table:**

[Co_2_F_2_(C_21_H_24_N_4_)_2_](BF_4_)_2_	*Z* = 1
*M**_r_* = 994.36	*F*(000) = 510.00
Triclinic, *P*1	*D*_x_ = 1.554 Mg m^−^^3^
*a* = 8.7884 (17) Å	Mo *K*α radiation, λ = 0.71075 Å
*b* = 11.334 (4) Å	Cell parameters from 1627 reflections
*c* = 11.897 (2) Å	θ = 3.0–27.5°
α = 64.91 (5)°	µ = 0.87 mm^−^^1^
β = 82.04 (6)°	*T* = 120 K
γ = 87.98 (7)°	Platelet, purple
*V* = 1062.5 (7) Å^3^	0.20 × 0.20 × 0.10 mm

### Data collection

**Table:**

Rigaku Mercury70 diffractometer	3660 reflections with *F*^2^ > 2σ(*F*^2^)
Detector resolution: 7.314 pixels mm^-1^	*R*_int_ = 0.033
ω scans	θ_max_ = 27.5°, θ_min_ = 3.0°
Absorption correction: multi-scan (*REQAB*; Rigaku, 1998)	*h* = −9→11
*T*_min_ = 0.787, *T*_max_ = 0.917	*k* = −14→14
8221 measured reflections	*l* = −12→15
4623 independent reflections	

### Refinement

**Table:**

Refinement on *F*^2^	Secondary atom site location: difference Fourier map
*R*[*F*^2^ > 2σ(*F*^2^)] = 0.053	Hydrogen site location: inferred from neighbouring sites
*wR*(*F*^2^) = 0.157	H-atom parameters constrained
*S* = 1.01	*w* = 1/[σ^2^(*F*_o_^2^) + (0.1*P*)^2^] where *P* = (*F*_o_^2^ + 2*F*_c_^2^)/3
4623 reflections	(Δ/σ)_max_ < 0.001
289 parameters	Δρ_max_ = 0.98 e Å^−^^3^
0 restraints	Δρ_min_ = −0.62 e Å^−^^3^
Primary atom site location: structure-invariant direct methods	

### Special details

**Table:**

Refinement. Refinement was performed using all reflections. The weighted *R*-factor (*wR*) and goodness of fit (*S*) are based on *F*^2^. *R*-factor (gt) are based on *F*. The threshold expression of *F*^2^ > 2.0 σ(*F*^2^) is used only for calculating *R*-factor (gt).

### Fractional atomic coordinates and isotropic or equivalent isotropic displacement parameters (Å^2^)

**Table:**

	*x*	*y*	*z*	*U*_iso_*/*U*_eq_	
Co1	0.03362 (4)	0.54291 (4)	0.10441 (3)	0.01641 (16)	
F1	−0.14216 (19)	0.48323 (17)	0.05164 (15)	0.0185 (4)	
F2	0.4169 (3)	0.7066 (3)	0.4284 (2)	0.0545 (7)	
F3	0.3216 (3)	0.8992 (3)	0.3089 (3)	0.0720 (10)	
F4	0.5502 (3)	0.8964 (3)	0.3775 (2)	0.0501 (6)	
F5	0.5324 (3)	0.8204 (3)	0.2337 (2)	0.0598 (8)	
N1	0.1242 (3)	0.3500 (3)	0.2284 (2)	0.0201 (6)	
N2	−0.0424 (3)	0.5803 (2)	0.2650 (2)	0.0163 (5)	
N3	0.2518 (3)	0.5991 (3)	0.1259 (2)	0.0196 (5)	
N4	0.0363 (3)	0.7600 (3)	−0.0106 (2)	0.0200 (5)	
C1	−0.2383 (4)	0.7885 (4)	−0.0223 (3)	0.0269 (7)	
C2	−0.0771 (4)	0.8417 (3)	−0.0566 (3)	0.0240 (7)	
C3	−0.0463 (5)	0.9744 (3)	−0.1339 (3)	0.0323 (8)	
C4	0.1020 (5)	1.0247 (4)	−0.1588 (3)	0.0355 (9)	
C5	0.2178 (5)	0.9412 (4)	−0.1059 (3)	0.0328 (8)	
C6	0.1820 (4)	0.8104 (3)	−0.0355 (3)	0.0236 (7)	
C7	0.3070 (4)	0.7150 (3)	0.0098 (3)	0.0258 (7)	
C8	0.2372 (3)	0.6245 (3)	0.2389 (3)	0.0217 (7)	
C9	0.0739 (3)	0.6280 (3)	0.2991 (3)	0.0173 (6)	
C10	0.0500 (4)	0.6739 (3)	0.3904 (3)	0.0218 (7)	
C11	−0.0988 (4)	0.6703 (3)	0.4504 (3)	0.0237 (7)	
C12	−0.2171 (4)	0.6211 (3)	0.4182 (3)	0.0243 (7)	
C13	−0.1872 (4)	0.5767 (3)	0.3248 (3)	0.0192 (6)	
C14	−0.3157 (4)	0.5238 (3)	0.2890 (3)	0.0246 (7)	
C15	0.3529 (3)	0.4882 (3)	0.1383 (3)	0.0225 (7)	
C16	0.2771 (4)	0.3608 (3)	0.2283 (3)	0.0228 (7)	
C17	0.3622 (4)	0.2584 (4)	0.3017 (3)	0.0317 (8)	
C18	0.2910 (5)	0.1365 (4)	0.3696 (4)	0.0367 (9)	
C19	0.1369 (5)	0.1239 (4)	0.3671 (3)	0.0319 (8)	
C20	0.0532 (4)	0.2333 (3)	0.2997 (3)	0.0247 (7)	
C21	−0.1182 (4)	0.2238 (4)	0.3075 (3)	0.0296 (8)	
B1	0.4541 (5)	0.8333 (4)	0.3389 (4)	0.0308 (9)	
H1A	−0.29767	0.83787	−0.09082	0.0323*	
H1B	−0.28508	0.79605	0.05397	0.0323*	
H1C	−0.23792	0.69658	−0.00753	0.0323*	
H3	−0.12718	1.02968	−0.1692	0.0388*	
H4	0.12426	1.11443	−0.21091	0.0426*	
H5	0.31982	0.97358	−0.11792	0.0394*	
H7A	0.348	0.68666	−0.05641	0.0309*	
H7B	0.39202	0.7586	0.02541	0.0309*	
H8A	0.28931	0.70915	0.21639	0.0261*	
H8B	0.2925	0.5563	0.30206	0.0261*	
H10	0.13352	0.7073	0.41203	0.0261*	
H11	−0.11794	0.7019	0.51321	0.0284*	
H12	−0.31878	0.61724	0.45919	0.0292*	
H14A	−0.27778	0.5055	0.21678	0.0295*	
H14B	−0.39692	0.58799	0.26711	0.0295*	
H14C	−0.35706	0.44322	0.35962	0.0295*	
H15A	0.38062	0.48718	0.05522	0.0270*	
H15B	0.44895	0.49994	0.16765	0.0270*	
H17	0.46723	0.27156	0.30536	0.0381*	
H18	0.34769	0.06351	0.41683	0.0440*	
H19	0.08654	0.04083	0.411	0.0383*	
H21A	−0.14588	0.24863	0.22358	0.0355*	
H21B	−0.16664	0.28245	0.34239	0.0355*	
H21C	−0.15395	0.13398	0.36185	0.0355*	

### Atomic displacement parameters (Å^2^)

**Table:**

	*U*^11^	*U*^22^	*U*^33^	*U*^12^	*U*^13^	*U*^23^
Co1	0.0172 (3)	0.0196 (3)	0.0131 (2)	−0.00145 (16)	−0.00164 (15)	−0.00753 (17)
F1	0.0178 (9)	0.0233 (9)	0.0151 (8)	−0.0020 (7)	−0.0006 (7)	−0.0092 (7)
F2	0.0382 (13)	0.0528 (16)	0.0428 (14)	−0.0055 (12)	−0.0063 (11)	0.0091 (12)
F3	0.0525 (16)	0.0447 (16)	0.138 (3)	0.0222 (13)	−0.0531 (18)	−0.0473 (19)
F4	0.0437 (14)	0.0678 (18)	0.0561 (16)	−0.0020 (13)	−0.0153 (12)	−0.0399 (14)
F5	0.0763 (19)	0.0585 (17)	0.0446 (15)	−0.0296 (14)	0.0170 (13)	−0.0271 (13)
N1	0.0242 (14)	0.0230 (14)	0.0158 (12)	0.0022 (11)	−0.0044 (10)	−0.0106 (11)
N2	0.0175 (13)	0.0184 (13)	0.0122 (11)	0.0012 (10)	−0.0011 (9)	−0.0061 (10)
N3	0.0186 (13)	0.0256 (14)	0.0151 (12)	−0.0026 (11)	−0.0009 (10)	−0.0092 (11)
N4	0.0280 (14)	0.0176 (13)	0.0139 (12)	0.0008 (11)	−0.0009 (10)	−0.0068 (10)
C1	0.0287 (18)	0.0301 (19)	0.0240 (16)	0.0063 (14)	−0.0054 (14)	−0.0133 (14)
C2	0.0362 (19)	0.0224 (16)	0.0147 (14)	0.0004 (14)	−0.0031 (13)	−0.0093 (13)
C3	0.052 (2)	0.0202 (17)	0.0215 (16)	0.0070 (16)	−0.0100 (16)	−0.0050 (14)
C4	0.058 (3)	0.0219 (18)	0.0235 (17)	−0.0070 (17)	−0.0060 (17)	−0.0056 (14)
C5	0.044 (2)	0.0263 (18)	0.0262 (17)	−0.0134 (16)	0.0015 (15)	−0.0098 (15)
C6	0.0300 (18)	0.0227 (16)	0.0174 (15)	−0.0062 (14)	−0.0015 (13)	−0.0079 (13)
C7	0.0214 (17)	0.0293 (18)	0.0220 (16)	−0.0110 (14)	0.0033 (13)	−0.0074 (14)
C8	0.0177 (16)	0.0324 (18)	0.0216 (15)	−0.0040 (13)	−0.0036 (12)	−0.0171 (14)
C9	0.0209 (16)	0.0169 (14)	0.0128 (13)	0.0016 (12)	−0.0039 (11)	−0.0046 (11)
C10	0.0265 (17)	0.0226 (16)	0.0177 (15)	0.0002 (13)	−0.0062 (12)	−0.0090 (13)
C11	0.0320 (18)	0.0241 (17)	0.0186 (15)	0.0064 (14)	−0.0064 (13)	−0.0121 (13)
C12	0.0243 (17)	0.0288 (18)	0.0160 (15)	0.0042 (14)	−0.0017 (12)	−0.0064 (13)
C13	0.0214 (16)	0.0177 (15)	0.0156 (14)	0.0052 (12)	−0.0025 (12)	−0.0048 (12)
C14	0.0203 (16)	0.0320 (18)	0.0214 (16)	−0.0021 (14)	0.0014 (12)	−0.0122 (14)
C15	0.0153 (15)	0.0328 (18)	0.0263 (16)	0.0043 (13)	−0.0063 (12)	−0.0184 (14)
C16	0.0266 (17)	0.0280 (17)	0.0189 (15)	0.0066 (14)	−0.0072 (13)	−0.0142 (13)
C17	0.032 (2)	0.040 (2)	0.0297 (18)	0.0147 (17)	−0.0132 (15)	−0.0198 (17)
C18	0.049 (2)	0.036 (2)	0.0314 (19)	0.0254 (19)	−0.0175 (17)	−0.0188 (17)
C19	0.052 (2)	0.0240 (18)	0.0195 (16)	0.0086 (17)	−0.0076 (16)	−0.0090 (14)
C20	0.0370 (19)	0.0230 (17)	0.0130 (14)	0.0015 (14)	−0.0036 (13)	−0.0066 (13)
C21	0.039 (2)	0.0282 (18)	0.0187 (16)	−0.0073 (15)	0.0009 (14)	−0.0081 (14)
B1	0.027 (2)	0.034 (2)	0.035 (2)	0.0009 (17)	−0.0083 (17)	−0.0169 (18)

### Geometric parameters (Å, º)

**Table:**

Co1—F1	1.985 (2)	C8—H8B	0.990
Co1—F1^i^	2.098 (2)	C10—H10	0.950
Co1—N1	2.249 (3)	C11—H11	0.950
Co1—N2	2.143 (3)	C12—H12	0.950
Co1—N3	2.124 (3)	C14—H14A	0.980
Co1—N4	2.251 (3)	C14—H14B	0.980
C1—H1A	0.980	C14—H14C	0.980
C1—H1B	0.980	C15—H15A	0.990
C1—H1C	0.980	C15—H15B	0.990
C3—H3	0.950	C17—H17	0.950
C4—H4	0.950	C18—H18	0.950
C5—H5	0.950	C19—H19	0.950
C7—H7A	0.990	C21—H21A	0.980
C7—H7B	0.990	C21—H21B	0.980
C8—H8A	0.990	C21—H21C	0.980
			
C2—C1—H1A	109.475	C11—C12—H12	120.262
C2—C1—H1B	109.475	C13—C12—H12	120.262
C2—C1—H1C	109.470	C13—C14—H14A	109.477
H1A—C1—H1B	109.471	C13—C14—H14B	109.471
H1A—C1—H1C	109.471	C13—C14—H14C	109.474
H1B—C1—H1C	109.464	H14A—C14—H14B	109.469
C2—C3—H3	120.021	H14A—C14—H14C	109.471
C4—C3—H3	120.022	H14B—C14—H14C	109.466
C3—C4—H4	120.711	N3—C15—H15A	109.251
C5—C4—H4	120.709	N3—C15—H15B	109.254
C4—C5—H5	120.552	C16—C15—H15A	109.247
C6—C5—H5	120.552	C16—C15—H15B	109.257
N3—C7—H7A	109.158	H15A—C15—H15B	107.925
N3—C7—H7B	109.159	C16—C17—H17	120.594
C6—C7—H7A	109.163	C18—C17—H17	120.604
C6—C7—H7B	109.167	C17—C18—H18	120.738
H7A—C7—H7B	107.874	C19—C18—H18	120.746
N3—C8—H8A	108.359	C18—C19—H19	119.864
N3—C8—H8B	108.356	C20—C19—H19	119.859
C9—C8—H8A	108.352	C20—C21—H21A	109.472
C9—C8—H8B	108.358	C20—C21—H21B	109.469
H8A—C8—H8B	107.440	C20—C21—H21C	109.469
C9—C10—H10	120.591	H21A—C21—H21B	109.475
C11—C10—H10	120.590	H21A—C21—H21C	109.474
C10—C11—H11	120.224	H21B—C21—H21C	109.468
C12—C11—H11	120.224		
			
F1—Co1—F1^i^—Co1^i^	0.00 (8)	C16—N1—C20—C21	175.4 (3)
F1^i^—Co1—F1—Co1^i^	−0.00 (9)	C20—N1—C16—C15	174.2 (3)
F1—Co1—N1—C16	151.42 (17)	C20—N1—C16—C17	−2.5 (5)
F1—Co1—N1—C20	−29.6 (3)	Co1—N2—C9—C8	−10.8 (3)
N1—Co1—F1—Co1^i^	−86.50 (11)	Co1—N2—C9—C10	172.26 (16)
F1—Co1—N2—C9	−176.69 (11)	Co1—N2—C13—C12	−171.21 (15)
F1—Co1—N2—C13	−4.6 (2)	Co1—N2—C13—C14	8.8 (3)
N2—Co1—F1—Co1^i^	179.51 (7)	C9—N2—C13—C12	0.4 (3)
F1—Co1—N4—C2	28.6 (3)	C9—N2—C13—C14	−179.6 (2)
F1—Co1—N4—C6	−150.43 (17)	C13—N2—C9—C8	176.1 (2)
N4—Co1—F1—Co1^i^	87.98 (12)	C13—N2—C9—C10	−0.8 (4)
F1^i^—Co1—N1—C16	73.10 (19)	Co1—N3—C7—C6	42.6 (3)
F1^i^—Co1—N1—C20	−107.9 (3)	Co1—N3—C8—C9	−9.8 (3)
N1—Co1—F1^i^—Co1^i^	100.85 (12)	Co1—N3—C15—C16	−46.2 (3)
F1^i^—Co1—N3—C7	60.13 (17)	C7—N3—C8—C9	109.5 (3)
F1^i^—Co1—N3—C8	−177.16 (14)	C8—N3—C7—C6	−78.0 (3)
F1^i^—Co1—N3—C15	−57.08 (13)	C7—N3—C15—C16	−161.6 (3)
N3—Co1—F1^i^—Co1^i^	179.26 (9)	C15—N3—C7—C6	157.0 (3)
F1^i^—Co1—N4—C2	107.2 (3)	C8—N3—C15—C16	72.6 (3)
F1^i^—Co1—N4—C6	−71.84 (18)	C15—N3—C8—C9	−126.4 (2)
N4—Co1—F1^i^—Co1^i^	−102.42 (12)	Co1—N4—C2—C1	5.2 (5)
N1—Co1—N2—C9	82.20 (16)	Co1—N4—C2—C3	−175.7 (2)
N1—Co1—N2—C13	−105.7 (2)	Co1—N4—C6—C5	179.1 (2)
N2—Co1—N1—C16	−98.0 (2)	Co1—N4—C6—C7	3.4 (4)
N2—Co1—N1—C20	81.0 (3)	C2—N4—C6—C5	−0.1 (5)
N1—Co1—N3—C7	149.19 (19)	C2—N4—C6—C7	−175.8 (3)
N1—Co1—N3—C8	−88.09 (16)	C6—N4—C2—C1	−175.9 (3)
N1—Co1—N3—C15	31.99 (13)	C6—N4—C2—C3	3.3 (5)
N3—Co1—N1—C16	−14.90 (18)	N4—C2—C3—C4	−3.3 (6)
N3—Co1—N1—C20	164.1 (3)	C1—C2—C3—C4	175.8 (3)
N1—Co1—N4—C2	−165.2 (2)	C2—C3—C4—C5	0.1 (6)
N1—Co1—N4—C6	15.7 (4)	C3—C4—C5—C6	3.0 (6)
N4—Co1—N1—C16	−14.8 (4)	C4—C5—C6—N4	−3.1 (6)
N4—Co1—N1—C20	164.1 (2)	C4—C5—C6—C7	172.5 (3)
N2—Co1—N3—C7	−119.29 (18)	N4—C6—C7—N3	−31.1 (5)
N2—Co1—N3—C8	3.42 (14)	C5—C6—C7—N3	153.1 (3)
N2—Co1—N3—C15	123.50 (14)	N3—C8—C9—N2	14.5 (3)
N3—Co1—N2—C9	3.94 (14)	N3—C8—C9—C10	−168.5 (2)
N3—Co1—N2—C13	175.99 (19)	N2—C9—C10—C11	0.4 (4)
N2—Co1—N4—C2	−81.5 (3)	C8—C9—C10—C11	−176.5 (2)
N2—Co1—N4—C6	99.44 (19)	C9—C10—C11—C12	0.4 (4)
N4—Co1—N2—C9	−74.66 (17)	C10—C11—C12—C13	−0.8 (4)
N4—Co1—N2—C13	97.4 (2)	C11—C12—C13—N2	0.4 (4)
N3—Co1—N4—C2	−165.2 (3)	C11—C12—C13—C14	−179.6 (2)
N3—Co1—N4—C6	15.79 (17)	N3—C15—C16—N1	36.2 (5)
N4—Co1—N3—C7	−30.78 (17)	N3—C15—C16—C17	−147.1 (3)
N4—Co1—N3—C8	91.93 (17)	N1—C16—C17—C18	6.0 (6)
N4—Co1—N3—C15	−147.99 (15)	C15—C16—C17—C18	−170.5 (3)
Co1—N1—C16—C15	−6.7 (4)	C16—C17—C18—C19	−3.7 (6)
Co1—N1—C16—C17	176.6 (3)	C17—C18—C19—C20	−1.7 (6)
Co1—N1—C20—C19	177.9 (2)	C18—C19—C20—N1	5.4 (6)
Co1—N1—C20—C21	−3.5 (5)	C18—C19—C20—C21	−173.2 (4)
C16—N1—C20—C19	−3.2 (5)		

Symmetry code: (i) −*x*, −*y*+1, −*z*.

### Hydrogen-bond geometry (Å, º)

**Table:**

*D*—H···*A*	*D*—H	H···*A*	*D*···*A*	*D*—H···*A*
C1—H1*C*···F1	0.98	2.37	3.298 (5)	157
C3—H3···F3^ii^	0.95	2.43	3.296 (5)	152
C5—H5···F5^iii^	0.95	2.46	3.231 (6)	139
C10—H10···F2	0.95	2.52	3.372 (5)	149
C10—H10···F3	0.95	2.53	3.294 (5)	138
C12—H12···F2^iv^	0.95	2.51	3.327 (5)	143
C14—H14*A*···F1	0.98	2.26	3.220 (4)	167
C14—H14*C*···F2^v^	0.98	2.36	3.287 (4)	157

Symmetry codes: (ii) −*x*, −*y*+2, −*z*; (iii) −*x*+1, −*y*+2, −*z*; (iv) *x*−1, *y*, *z*; (v) −*x*, −*y*+1, −*z*+1.
