# Supplementary material for: Crystal structure of a dinuclear CoII complex with bridging fluoride ligands: di-μ-fluorido-bis­{tris­[(6-methyl­pyridin-2-yl)meth­yl]amine}­dicobalt(II) bis­(tetra­fluorido­borate)
Source: Acta Crystallogr Sect E Struct Rep Online. 2014 Oct 4;70(Pt 11):m359–60. doi: 10.1107/S1600536814021631 (PMC4257317; doi:10.1107/S1600536814021631)
Supplement: Supplementary file 3 [file e-70-0m359-Isup3.doc]

**[Supplementary materials]**

**Crystal structure of dinuclear Co complex with bridging fluorides: μ-difluoro-*bis*{**

***tris*(6-methyl-2-pyridylmethyl)amine}dicobalt(II) tetrafluoroborate, [Co_2_(Me_3_tpa)_2_F_2_](BF_4_)_2_**

**Masataka Inomata*a*, Yusaku Suenaga*a****

1. **Comment**

Reaction with Co(BF_4_)_2_・6H_2_O and Me_3_tpa (*tris*(6-methyl-2-pyridylmethyl)amine) in dry methanol at ambient temperature for 7 days produced a purple crystals of [Co_2_(Me_3_tpa)_2_F_2_](BF_4_)_2_ in 18 % yield. Characterization was on the basis of IR, a satisfactory elemental analysis and single crystal crystallographic data. X-ray diffraction data was collected at 120 K to improve the low diffracting power of the crystal. Crystallographic data is presented in Table 1 and a view of the complex cation is shown in Figure 1. The two cobalt ions are six-coordinate by fluoride in a octahedral coordination, and each of tripodal ligand adopts a folded conformation around the metal ion. The Co-N bond lengths are 2.248(4), 2.143(3), 2.122(3) and 2.248(3) Å. These indicate the high-spin Co^II^ state at 150 K as the bond lengths is similar to reported Co-N (2.295(10), 2.165(9), 2.091(9), 2.212(10) Å) values for high-spin Co^II^. In concerning fluoro-bridged dimeric structure, the decomposition of the BF_4_^-^ ion occurs, yeilding F^-^ and as resulting two cobalt ions are bridged by fluoro ions.

Solid state magnetic measurements for polycrystalline sample of [Co_2_(Me_3_tpa)_2_F_2_](BF_4_)_2_ were investigated using a SQUID magnetometer over the temperature range 2-300 K using an external field of 10 kOe. The room temperature value of *χ_M_T* is 6.76 cm^3^Kmol^-1^, which is higher than spin-only value of 3.75 cm^3^Kmol^-1^ calculated for two non-interacting high spin cobalt(II) ion (S=3/2). This may be due to distorted high-spin octahedral cobalt(II) ions and intermolecular interaction through π...π. When the temperature is lowered, the *χ_M_T* value decreases slightly to 0.33 cm^3^Kmol^-1^ at 2.0 K.

1. **Experimental**

A solution of Co(BF_4_)_2_・6H_2_O (204 mg, 0.60 mmol) in dry methanol (20 mL) was added to a methanol solution (20 mL) of *tris*(6-methyl-2-pyridylmethyl)amine (199 mg, 0.60 mmol). The resulting solution was stirred for 1 hr. Diethylether was added to the filtrate slowly to obtain the complex. This solution stand at ambient temperature and over the period of 7 days a purple microcrystals of [Co_2_(Me_3_tpa)_2_F_2_](BF_4_)_2_ separated from the solution in 18 % (107 mg) yield. IR (KBr, cm^-1^); 3448s, 1605s, 1578m, 1451s, 1352m, 1084s, 789m, 522m, ESI-MS; *m/z*=907.26(M-BF_4_), Anal. Calc. for C_42_H_48_N_8_F_2_Co_2_B_2_F_8_: C, 50.73; H, 4.87; N, 11.27%. Found: C, 50.96; H, 4.63; N, 11.07%.

**3. Refinement**

Hydrogen atoms were refined using the rising model. The final cycle of full-matrix least-squares refinement on F^2^ was based on 4623 observed reflections and 289 variable parameters and converged (largest parameters shift was 0.00 times its esd) with unweighted and weighted agreement factors of R1=0.0527 and wR2=0.1569. The standard deviation of an observation of unit weights were used. The maximum and minimum peaks on the final difference Fourier map corresponded to 0.98 and -0.62 e^-^/ Å^3^, respectively.

**Computing details**

Data collection: [*CrystalClear* (Rigaku Inc., 2008)](../20130729-Co-Me3tpa-BF4-MeOH%20_computing_data_collection); cell refinement: [*CrystalClear*](../20130729-Co-Me3tpa-BF4-MeOH%20_computing_cell_refinement); data reduction: [*CrystalClear*](../20130729-Co-Me3tpa-BF4-MeOH%20_computing_data_reduction); program(s) used to solve structure: [Il Milione (Burla, *et al.*, 2007)](../20130729-Co-Me3tpa-BF4-MeOH%20_computing_structure_solution); program(s) used to refine structure: [*SHELXL97* (Sheldrick, 2008)](../20130729-Co-Me3tpa-BF4-MeOH%20_computing_structure_refinement); molecular graphics: [*CrystalStructure* 4.0](../20130729-Co-Me3tpa-BF4-MeOH%20_computing_molecular_graphics); software used to prepare material for publication: [*CrystalStructure* 4.0 (Rigaku, 2010)](../20130729-Co-Me3tpa-BF4-MeOH%20_computing_publication_material).

**References**

Kralingen, C. G., Reedijk, J. (1976). *J. C. S. Chem. Commun*., 533.

Beni, A., Dei, A., Laschi, S., Rizzitano, M., Sorace, L. (2008). *Chem. Eur. J.*, 14, 1804-1813.

Ding, K., Dugan, T. R., Brennessel, W. W., Bill, E., Holland, P. L. (2009). *Organometallics,* 28, 6650-6656.

Dugan, T. R., Goldberg, J. M., Brennessel, W. W., Bill, E., Holland, P. L. (2012). *Organometallics,* 31, 1349-1360.

Massoud, S. S., Broussard, K. T., Mautner, F. A., Vicente, R., Saha, M. K., Bernal, I. (2008). *Inorg. Chim. Acta*, 361, 123-131.

Zhu, Q., Nelson, K. J., Shum, W. W., Dipasquale, A., Rheingold, A. L., Miller, J. S. (2009). *Inorg. Chim. Acta*, 362, 595-598.
