# Supplementary figures and images for: Crystal structure of a dinuclear CoII complex with bridging fluoride ligands: di-μ-fluorido-bis­{tris­[(6-methyl­pyridin-2-yl)meth­yl]amine}­dicobalt(II) bis­(tetra­fluorido­borate)
Source: Acta Crystallogr Sect E Struct Rep Online. 2014 Oct 4;70(Pt 11):m359–60. doi: 10.1107/S1600536814021631 (PMC4257317; doi:10.1107/S1600536814021631)

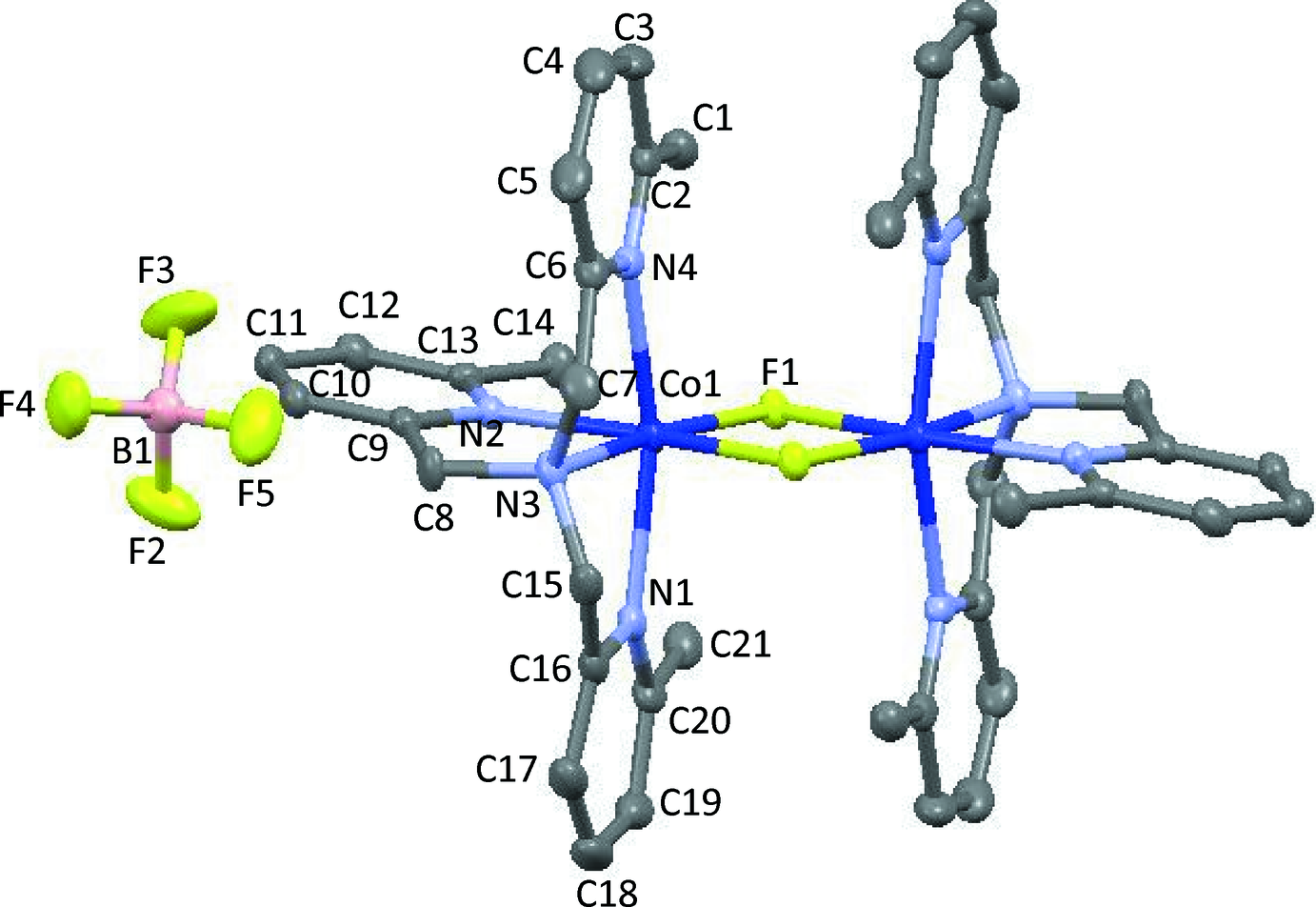

Supplement: Supplementary file 4 [file e-70-0m359-fig1.tif]
